# A New Generation of Fibers Based on Yb^3+^-Doped Crystals Embedded in Phosphate Glass

**DOI:** 10.1021/acsomega.4c10954

**Published:** 2025-04-17

**Authors:** Natalia Vakula, Matiss Bardins, Khaldoon Nasser, Bartosz Bondzior, Catherine Boussard-Plédel, Johann Troles, Laeticia Petit

**Affiliations:** † Photonics Laboratory, Tampere University, Korkeakoulunkatu 3, Tampere 33720, Finland; ‡ Institute of Low Temperature and Structure Research, Polish Academy of Sciences, Okólna 2, Wroclaw 50-422, Poland; § Univ Rennes, CNRS, ISCR [(Institut des Sciences Chimiques de Rennes)], UMR 6226, Rennes F-35000, France

## Abstract

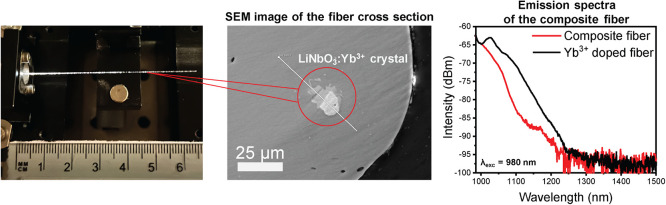

Composites, defined here as crystals embedded in glass matrices, are promising materials for photonics applications, as they combine the properties of the crystals with the beneficial properties of glass (easy and cheap to process, drawing capability). Yb^3+^-ion doping is of particular interest for developing laser fibers emitting around 1 μm due to its simple energy levels, minimal excited-state absorption, and reduced quenching effects. Here, CaWO_4_:Yb^3+^, Yb_2_Si_2_O_7_, and LiNbO_3_:Yb^3+^ crystals are prepared using a solid-state reaction and added into a phosphate glass matrix, the composition of which is selected based on its drawability. The spectroscopic properties of the composites are used to evidence the reaction of the crystals with the glass matrix during composite preparation. Partial to almost complete decomposition of the crystals occurs, depending on their composition and the composite preparation method, leading to the diffusion of the crystal elements into the glass network. The composite with embedded LiNbO_3_:Yb^3+^ crystals is successfully prepared as a glass-based rod, which is drawn into a single-index fiber. Despite the presence of crystals in the fiber, light can propagate through the fiber, which emits light at ∼1 μm. This study demonstrates the potential of composite fibers for lasing applications.

## Introduction

Due to its simple energy diagram with a single excited level (^2^F_5/2_), the Yb^3+^ ion has been of great interest, especially when introduced into a glass fiber. The resulting fibers can be used as fiber lasers and amplifiers for applications at ∼1 μm.^1^ However, for such applications, high peak power and pulse energies are required.

Due to its transparency, high mechanical strength, and thermal robustness, silica glass is one of the most commonly used materials for optical fiber production, with the first commercial fibers already available since the 1960s.^2^ However, silica glass has multiple disadvantages, the main one being related to the low solubility of rare-earth (RE) ions in the silica network.^3^ Therefore, efforts have been focused on the development of new Yb^3+^-doped fibers with enhanced spectroscopic properties. One approach is to use other glass systems to allow the increase of the amount of Yb^3+^ without forming Yb–Yb clusters. Phosphate,^4^ germanate,^5^ and tellurite^6^ glasses have been successfully doped with large amount of Yb_2_O_3_. The other approach is to use crystals, as they are well-known for exhibiting superior properties compared to glasses, such as transparency, absorption and emission cross-sections, and nonradiative relaxation rates when engineered with a proper crystalline phase. For example, CaWO_4_ crystal has emerged as an interesting crystal due to its large bandgap.^7^ Subbotin et al. demonstrated that this crystal, when doped with Yb^3+^, is promising as a down-converter for solar cell application.^8^ Lithium niobate (LiNbO_3_) crystal is another well-known crystal and has been widely investigated for its electrical and nonlinear optical properties.^9,10^ Excellent spectroscopic properties of Yb^3+^-doped LiNbO_3_ crystal have been reported by Boudour.^11^ Finally, Yb_2_Si_2_O_7_ crystal has been also of interest due to its phase stability across a large range of temperatures.^12^ This crystal can find applications as a scintillator with near-infrared (NIR) emission, as demonstrated by Horiai et al., who reported NIR emissions under X-ray excitation.^13^ However, there have been limited studies reported on fabricating fibers from these crystals due to the complexity of the growth and drawing processes. One method involves pulling the fiber from the melt,^14^ while another approach is laser-heated pedestal growth (LHPG).^15^ For example, a CO_2_ laser was used to melt a YAG rod from which a fiber was pulled upward.^16^

Therefore, effort has been focused on the development of crystals embedded in glass. The mostly used method to fabricate such material involves the *in situ* formation of crystals in the glass using thermal treatment.^17,18^ By selecting appropriate heat treatment parameters, such as temperature and duration, it is possible to control the nucleation and growth of the crystals.^19−21^ However, the thermal treatment should be tailored to control crystal growth. Indeed, to be considered a promising material for fiber drawing, the glass with embedded crystals should be transparent to limit light scattering, including Rayleigh scattering. The crystals should be much smaller than the excitation light’s wavelength to reduce Rayleigh scattering, and their size must be controlled to avoid Mie scattering. The separation between the crystals should be comparable to their size. Matching the refractive index of the crystals and the glass host is also important.^22^ The crystals should also be homogeneously distributed throughout the volume of the glass with no aggregation.^17^ This method faces multiple challenges, as clearly evidenced by Hongisto et al.^23^ For example, the growth of crystals in the volume of the glass at the expense of surface crystallization cannot be controlled. It is not possible to control either the phase of the crystals precipitating in the glass or the amount of RE ions in the crystals. The *in situ* growth of nanoparticles when applied to the fiber has additional drawbacks, such as the limited length of the fiber that can be heat-treated. The fiber needs to be stripped prior to the thermal treatment and then recoated.^24^

To overcome these limitations, another approach is to synthesize crystals with a specific crystalline phase and RE-dopant level and then to embed them in a glass matrix.^25^ This method allows for control over the spectroscopic properties of the RE ions, independent of the glass composition. However, one should be careful when adding the crystals to avoid their (partial to complete) decomposition in the glass network, as explained by Ojha et al.^26^ Indeed, when decomposition occurs, the crystal elements diffuse into the glass network, leading to changes in the RE sites in the crystals and, consequently, to changes in the crystals’ spectroscopic properties. Using this technology, various composites composed of glasses with embedded crystals have been developed.^27−32^ Composite fibers with embedded crystals were successfully obtained, demonstrating clearly the promise of this technology.^33,34^

Here, we demonstrate that CaWO_4_:Yb^3+^, Yb_2_Si_2_O_7_, and LiNbO_3_:Yb^3+^ crystals can be prepared using a solid-state reaction method and added into a phosphate glass matrix. These crystals were chosen for their high decomposition temperatures, enabling them to withstand the melting and fiber drawing processes.^35−37^ Aphosphate glass was selected as the glass host as it offers distinct advantages, such as easy processing, excellent thermo-mechanical and chemical properties, homogeneity, good thermal stability, and excellent optical properties, including high transparency in the UV–visible–near-infrared region.^38−41^ Phosphate glasses are known as ideal hosts for RE ions, primarily due to their high solubility for RE ions.^42^ Furthermore, these glasses containing more than 45 mol % P_2_O_5_ have been shown to be easily drawable into optical fibers.^33,43−45^ The spectroscopic and structural properties of the composites are measured in order to demonstrate the partial to complete decomposition of the crystals occurring during the melting and drawing processes. We demonstrate, for the first time to the best of our knowledge, the presence of LiNbO_3_:Yb^3+^ crystals in the as-drawn phosphate fiber.

## Experimental Section

### Reagents

CaWO_4_:Yb^3+^ crystals were prepared using WO_4_ (Honeywell-Fluka, 99%), CaCO_3_ (Alfa Aesar, 99%), Yb_2_O_3_ (REacton, 99.99%), and Na_2_CO_3_ (Alfa Aesar, 99.5%). LiNbO_3_:Yb^3+^ crystals were made using Nb_2_O_5_ (Thermo Fisher, 99.99%), Li_2_CO_3_ (Sigma-Aldrich, 99%), and Yb_2_O_3_ (REacton, 99.99%). Yb_2_Si_2_O_7_ crystals were synthesized using SiO_2_ (Umicore, 99.99%) and Yb_2_O_3_ (REacton, 99.99%). The glass was prepared using SrCO_3_ (Thermo Fisher, 97.5%), (NH_4_)_2_HPO_4_ (Thermo Fisher, 99%), and (NaPO_3_)_6_ (Thermo Fisher, tech).

### Crystals Synthesis Methods

The Ca_0.7_Na_0.15_Yb_0.15_WO_4_ (referred to as CaWO_4_:Yb^3+^ in the article) crystals were prepared via solid-state reaction. The substitution of trivalent dopants for divalent host ions Ca^2+^ was balanced by introducing monovalent Na^+^ ions in equal proportions to the trivalent dopants to maintain charge neutrality, as in the study of Nassau and Loiacono.^46^ The chemicals were heated in an alumina crucible from room temperature to 1200 °C using a 3 °C/min heating rate in an ambient atmosphere. The duration of thermal treatment at 1200 °C was 4 h, followed by gradual cooling.^47^

The Yb_2_Si_2_O_7_ crystals were synthesized using a solid state reaction in an alumina crucible, heated at 1600 °C for 5 h in an ambient atmosphere.^48^ The heating rate was 3 °C/min.

The initial synthesis of Li_0.975_Yb_0.025_NbO_3_ (referred to as LiNbO_3_:Yb^3+^ in the manuscript) followed the procedure outlined in Piva et al. study, developed to produce undoped LiNbO_3_.^49^ The temperature and duration of the heat treatment needed to be optimized in order to incorporate Yb^3+^ ions without forming a secondary phase (more information about the trials can be found in Figure S1). LiNbO_3_ with 2.5 at % of Yb^3+^ was successfully obtained using a thermal treatment at 1200 °C for 6 h with a 3 °C/min heating rate.

### Glass Synthesis Methods

Phosphate glass with the composition (in mol %) 50P_2_O_5_–40SrO–10Na_2_O was prepared using the standard melt-quench method. The glass batch was melted in a quartz crucible at 1050 °C for 30 min with a 15 °C/min heating rate. After being quenched, the glass was annealed at 400 °C for 6 h to release the stress caused by the quench.

### Composite Synthesis Methods

Composites were prepared using 2 different approaches (the flow diagram can be found in Figure S2). In the direct doping method (DDM), 2.5 wt % of crystals were added to the molten glass with the composition (in mol %) 50P_2_O_5_–40SrO–10Na_2_O melted at 1050 °C for 30 min. The glass melt was quickly and manually stirred and poured onto a steel plate for rapid quenching to form composite bulks. For composite preforms, the glassmelt was poured into a graphite mold that had been preheated to 300 °C. The composite bulks and preforms were then subjected to annealing at 400 °C for 6 and 17 h, respectively. After annealing, the composite preforms were polished and drawn into fibers at 580 °C under a helium gas flow of 1.5 L/min to maintain an inert atmosphere. No coating was applied during the drawing process, enabling measurement of the thermal and structural properties of the fibers.

Composites were also prepared by using the remelting process. The glass was first melted and then crushed into a powder using a mortar and pestle. The glass powder was mixed with 5 wt % of crystals. The mixture was melted at 950 °C for 7 min.^33^ After melting, the mixture was quenched by pouring it onto a steel plate, followed by annealing at 400 °C for 6 h.

### Characterization

A scanning electron microscope (SEM) (Crossbeam 540, Carl Zeiss, Oberkochen, Germany) equipped with an energy dispersive spectroscopy (EDS) detector (X-MaxN 80, Oxford Instruments, Abingdon-on-Thames, UK) was used to produce images and determine the composition of the samples. To minimize charging effects, the samples were coated with a conductive layer of carbon.

The crystal structures were analyzed using X-ray diffraction (XRD) patterns obtained with a PANalytical EMPYREAN multipurpose X-ray diffractometer (PANalytical, Almelo, The Netherlands). The measurements employed Ni-filtered Cu Kα radiation, and the diffractograms were recorded within an angular range of 2θ (15–90°), with a measurement step of 0.053°.

A UV–vis–near-IR spectrophotometer (UV-3600 Plus, Shimadzu) was used to measure the transmission spectra of polished glass samples (with a thickness of 1 cm) within the wavelength range of 200–1600 nm.

The emission spectra of the crystals and bulks were recorded in the range of 950–1200 nm using a spectrometer (iHR320, Jobin Yvon, Horiba Ltd., Kyoto, Japan) equipped with a detector (P4631-02, Hamamatsu Photonics K.K., Hamamatsu City, Japan). A 915 nm laser diode (K915FA3RN model) served as the excitation source. The measurements were taken from powder samples to facilitate a comparison of emission intensities between the samples. All spectroscopic measurements were carried out at room temperature.

The spectral properties of 3 cm-long optical fibers were measured using the fiber-pigtail white light source (MG922A, Anritsu) or a fiber-pigtail 980 nm laser source (Coherent). The spectral transmission of the fiber was measured from 600 to 1700 nm using the white light. The emission of the fiber samples was measured with a 980 nm laser source. Both light sources were attached to the fiber samples by the butt-coupling technique with multimode fibers. The output light was captured by another multimode fiber and analyzed with an optical spectrum analyzer (OSA), specifically the ANDO AQ6317B model.

The micro-Raman spectra were recorded using a Raman microscope (Renishaw inVia Qontor, Wotton-under-Edge, UK) in a backscattering configuration. The instrument utilized a 785 nm excitation laser focused onto the sample using a 50× objective and a 1200 lines/mm grating. Due to the nature of the samples, the crystallites were randomly oriented, leading to an averaging of vibrational contributions from different orientations. No specific polarization selection was applied during the measurements, as the random orientations of the grains inherently reduce polarization-dependent effects

## Results and Discussion

CaWO_4_:Yb^3+^, Yb_2_Si_2_O_7_, and LiNbO_3_:Yb^3+^ crystals were synthesized using the solid-state reaction method (see Experimental Section). In the CaWO_4_ crystal, the Yb^3+^ ions substitute the Ca^2+^ ions due to their similar ionic radii: 112 pm for Ca^2+^ and 98.5 pm for Yb^3+^ (in an 8-coordinate geometry).^54^ In the case of LiNbO_3_ crystals, the Yb^3+^ ions are expected to occupy the Nb^5+^ lattice site due to the smaller mismatch of ionic electronegativity between Yb^3+^ (1.438) and Nb^5+^ (1.479).^55,56^

**Figure 1:**
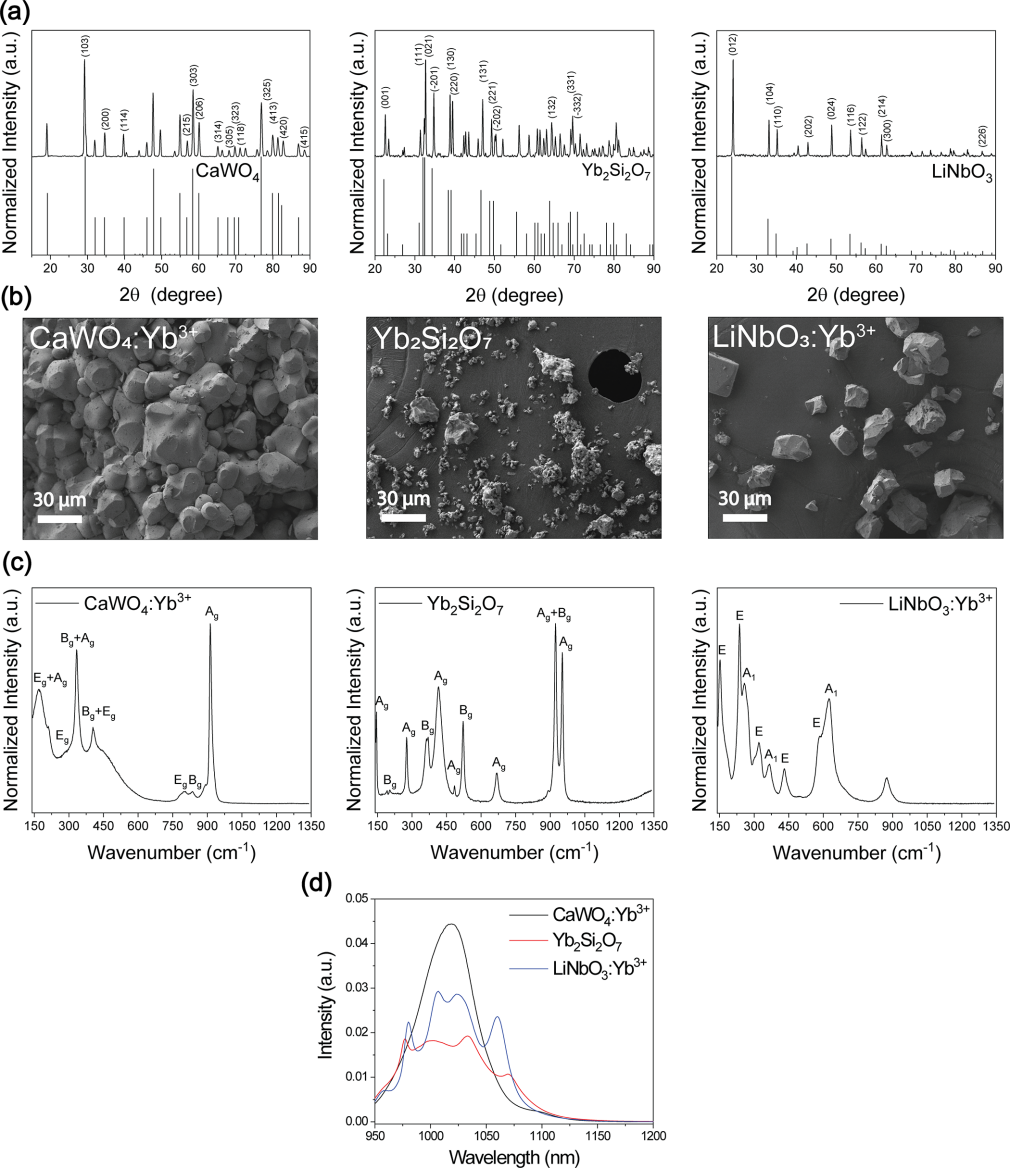
Structural analysis of the CaWO_4_:Yb^3+^, Yb_2_Si_2_O_7_, and LiNbO_3_:Yb^3+^ crystals. (a) XRD diffraction patterns of the as-prepared crystals compared to the standard powder diffraction files of CaWO_4_ (00-002-0619), Yb_2_Si_2_O_7_ (00-037-0458), and LiNbO_3_ (04-002-5061). (b) SEM images of the as-prepared CaWO_4_:Yb^3+^, Yb_2_Si_2_O_7_, and LiNbO_3_:Yb^3+^ crystals. (c) Micro-Raman spectrum of the as-prepared CaWO_4_:Yb^3+^, Yb_2_Si_2_O_7_, and LiNbO_3_:Yb^3+^ crystals with their band attribution according to Ferré et al.,^50^ Ogawa et al.,^51^ and Repelin et al.,^52^ respectively. Excitation wavelength is 785 nm. (d) Emission spectra of the as-prepared CaWO_4_:Yb^3+^, Yb_2_Si_2_O_7_, and LiNbO_3_:Yb^3+^ crystals under excitation at 915 nm.

The 3 crystals appear to be well crystallized, as evidenced by the sharp and well-defined diffraction peaks in the XRD patterns (Figure 1a). The XRD patterns of the crystals exhibit peaks corresponding to those of CaWO_4_, Yb_2_Si_2_O_7_, and LiNbO_3_ crystals.

The morphology and size of the CaWO_4_:Yb^3+^, Yb_2_Si_2_O_7_, and LiNbO_3_:Yb^3+^ crystals characterized using SEM (Figure 1b) show that the crystals are small granules with various shapes, and their sizes range from 10 to 30 μm. The grains are expected to consist of aggregations of many small crystallites.

The micro-Raman spectra of the CaWO_4_:Yb^3+^, Yb_2_Si_2_O_7_, and LiNbO_3_:Yb^3+^ crystals presented in Figure 1c are similar to those reported by Ferré et al.,^50^ Ogawa et al.,^51^ and Repelin et al.,^52^ respectively.

The emission spectra of the crystals presented in Figure 1d show an emission band corresponding to the ^2^F_5/2_ → ^2^F_7/2_ transition of the Yb^3+^ ions. CaWO_4_:Yb^3+^ crystals exhibit a wide band centered at 1020 nm and have the highest emission, while the emission spectra of the Yb_2_Si_2_O_7_ and LiNbO_3_:Yb^3+^ crystals feature a few distinct subpeaks, confirming the incorporation of Yb^3+^ in the crystal lattice.^8,13,57^ The broad emission from the CaWO_4_:Yb^3+^ should be related to a large distribution of crystal field strengths when Yb^3+^ replace Ca^2+^ as discussed by Sani et al.^58^ The sharp emission peaks observed from the Yb_2_Si_2_O_7_ and LiNbO_3_:Yb^3+^ crystals suggest that Yb^3+^ ions occupy well-defined lattice sites.^13,57^

The composite glasses were fabricated by incorporating the as-prepared crystals into a phosphate glass matrix using two methods: the direct doping method (DDM) and the remelt method. In the DDM, the crystals are added into the molten glass prior to quenching, while in the remelt approach, the glass is ground into fine powder and then remelted with the crystals for few minutes at a lower temperature than the melting temperature. Details of the two synthesis methods are given in the Experimental Section.

**Figure 2:**
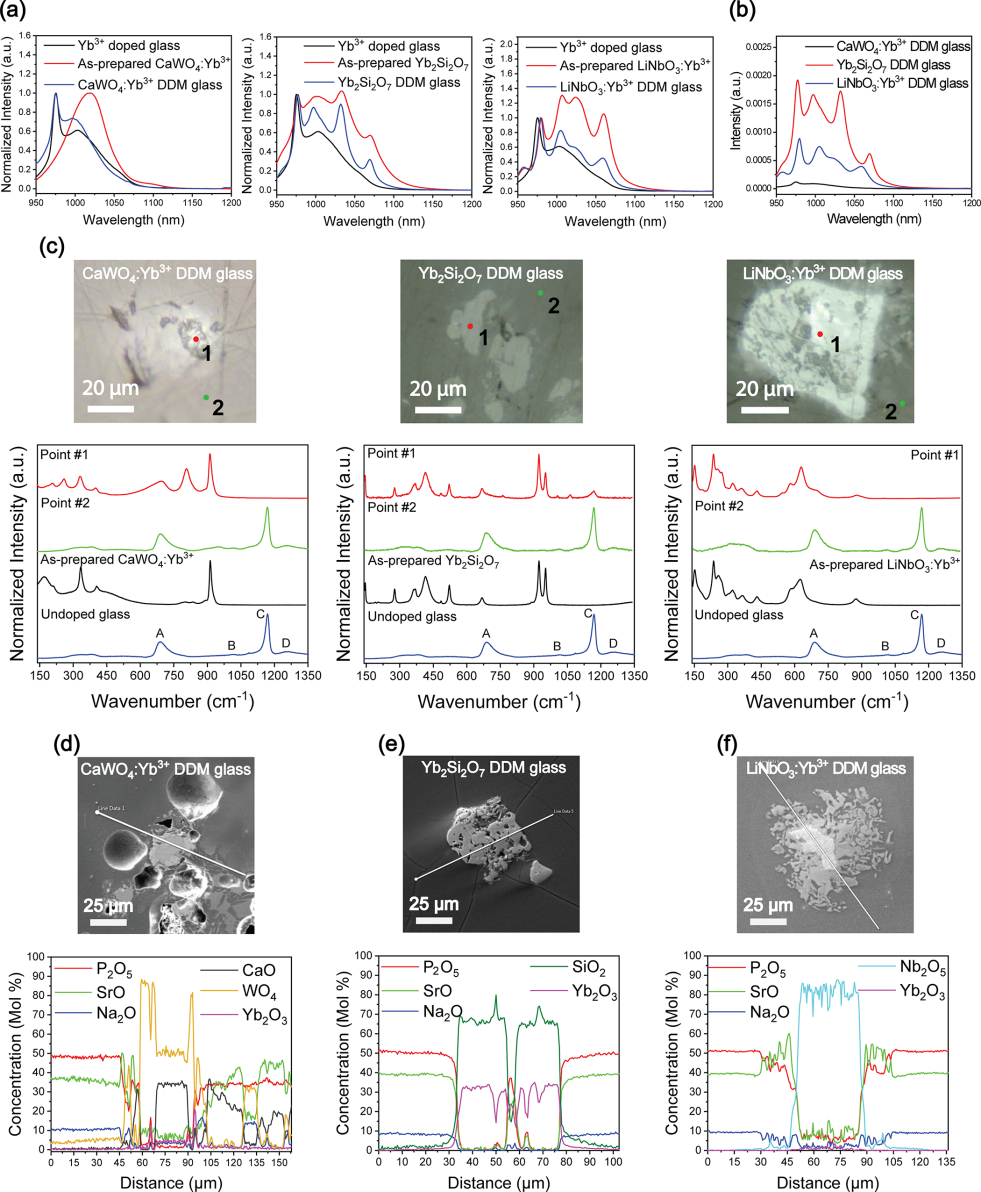
Structural and spectroscopic analysis of the DDM composites with embedded CaWO_4_:Yb^3+^, Yb_2_Si_2_O_7_, and LiNbO_3_:Yb^3+^ crystals. (a) Normalized emission spectrum of the DDM composites with embedded CaWO_4_:Yb^3+^, Yb_2_Si_2_O_7_, and LiNbO_3_:Yb^3+^ crystals under 915 nm excitation. (b) Intensity of the emission of the DDM composites with embedded CaWO_4_:Yb^3+^, Yb_2_Si_2_O_7_, and LiNbO_3_:Yb^3+^ crystals under 915 nm excitation. (c) Images and micro-Raman spectra of the CaWO_4_:Yb^3+^, Yb_2_Si_2_O_7_, and LiNbO_3_:Yb^3+^ crystals found at the surface of the composites. The complete Raman band attribution for the glass can be found in Massera et al.^53^ (d) SEM image and EDS line scan of a crystal found at the surface of the DDM composite with embedded CaWO_4_:Yb^3+^ crystals. (e) SEM image and EDS line scan of a crystal found at the surface of the DDM composite with embedded Yb_2_Si_2_O_7_ crystals. (f) SEM image and EDS line scan of a crystal found at the surface of the DDM composite with embedded LiNbO_3_:Yb^3+^ crystals.

Transparent composites were obtained using the remelt process, whereas the composites prepared using the DDM were opaque, suggesting that the fabrication method has an impact on the survival of the crystals during the melting process. It should be mentioned that translucent composites are expected due to the size of the crystals (30–40 μm) and the refractive index mismatch between the crystals ( at 589.3 nm,^59^ at 632.8 nm,^60^ at at 632.8 nm^61^) and the glass matrix (*n*_glass_ = 1.5 at 632.8 nm).^62,63^ Transparency might already suggest decomposition of the crystals during composite preparation. To confirm the presence of crystals in the composites, the emission spectra of the composites were measured and compared with those of the as-prepared crystals. The emission spectra of the composites prepared by the remelt technique show an emission band, which is similar to the emission band from standard Yb^3+^-doped glass, suggesting that the Yb^3+^ ions are located in amorphous sites. It is possible then to assume that the crystals did not survive this fabrication process (Figure S3a). On the other hand, most of the composites prepared using the DDM exhibit different emission bands than the Yb^3+^-doped glass (Figure 2a). Only the composite prepared with the CaWO_4_:Yb^3+^ crystals shows an emission band similar to that of Yb^3+^-doped glass, indicating that most of the CaWO_4_:Yb^3+^ crystals decompose during the composite fabrication process, leading to Yb^3+^ ions located in the glass matrix. Consequently, the composite prepared with this crystal exhibits the lowest intensity of emission compared to the 2 other composites (Figure 2b). Alternatively, the emission band from the composites with embedded Yb_2_Si_2_O_7_ and LiNbO_3_:Yb^3+^ crystals features several distinct subpeaks similar to those observed in the as-prepared crystals (Figure 1d), confirming that it is possible to embed the crystals in the glass matrix using DDM. Complete dissolution of crystals can then be avoided by adding the crystals into the glass melt prior to melting. A similar observation was reported when embedding SrAl_2_O_4_:Eu^2+^Dy^3+^ microparticles in the same glass matrix.^33^ It should be noted that the composite prepared with the Yb_2_Si_2_O_7_ crystals exhibits the highest intensity of emission.

The composites were polished, and their surfaces were checked using micro-Raman spectroscopy. The Raman spectra of undoped phosphate glass exhibit four primary bands: (A) ∼690 cm^–1^, (B) ∼1010 cm^–1^, (C) ∼1170 cm^–1^, and (D) ∼1250 cm^–1^ (Figure 2c). Band A corresponds to the symmetric P–O–P vibration in metaphosphate chains, while band B is attributed to the symmetric stretching mode of nonbridging oxygens (NBO) in Q_1_ units. Bands C and D correspond to the symmetric and antisymmetric vibrations of PO_2_ groups in phosphate chains, respectively.^53,64−66^ While no crystals are found on the surface of the composites prepared using the remelt method (Figure S3b), crystals are easily observed on the surface of the composites prepared using the DDM, confirming that the crystals did not dissolve completely during the composite preparation (Figure 2c). The micro-Raman spectra were collected from the crystals found on the surface of the composites in order to confirm the nature of the crystals. The micro-Raman spectra exhibit various peaks and bands that can be related not only to the 3 investigated crystals but also to the glass matrix. This confirms the survival not only of the Yb_2_Si_2_O_7_ and LiNbO_3_:Yb^3+^ crystals, as suspected from the spectroscopic properties of the composites, but also of some of the CaWO_4_:Yb^3+^ crystals during the composite preparation. It should be mentioned that the micro-Raman spectra collected from different areas on the surface of the composites prepared using the remelt technique are similar to the micro-Raman spectra of the glass. No micro-Raman peaks of the crystals could be detected in these composites, confirming that the crystals most likely dissolved completely into the glass matrix during the remelt process (Figure S3b).

Energy dispersive spectroscopy (EDS) composition analysis was performed to complete the micro-Raman analysis (Figure 2d–f). For the 3 composites, the composition of the glass matrix matches the theoretical one within a margin of ±1.5 mol %. However, up to 5 mol % of WO_4_ was detected in the glass part of the composite prepared with CaWO_4_:Yb^3+^, confirming the dissolution of some of the CaWO_4_:Yb^3+^ crystals during the composite preparation using the DDM. According to the line scan across a CaWO_4_:Yb^3+^ crystal, the remaining crystals in the composites are suspected to maintain their composition at their center. There is an outer layer rich in W, confirming that the crystals react with the glass matrix, leading to the diffusion of Ca (and maybe Yb) from the crystals into the glass matrix during the composite preparation (Figure 2d). Crystals can be seen at the CaWO_4_:Yb^3+^ and glass interface, the composition of which is Sr-rich phosphate. Similar crystals were reported when adding SrAl_2_O_4_:Eu^2+^Dy^3+^ particles to the same glass system.^26^

2 mol % of SiO_2_ was detected in the glass part of the composite prepared with the Yb_2_Si_2_O_7_ crystals, suggesting that Yb_2_Si_2_O_7_ crystals also dissolve during the composite preparation. From the composition analysis across a Yb_2_Si_2_O_7_ crystal found on the surface of the composite, these large crystals maintain their composition, suggesting that these crystals are stable in the glass with very limited interaction with the glass (Figure 2e). Finally, the composition analysis performed across a LiNbO_3_:Yb^3+^ crystal found on the surface of the composite is presented in Figure 2f. As observed in the composite with embedded CaWO_4_:Yb^3+^, the elements from the LiNbO_3_:Yb^3+^ crystals diffuse into the glass, leading to the precipitation of large Sr-rich phosphate crystals at the LiNbO_3_:Yb^3+^ crystal–glass interface. From the composition analysis, partial dissolution of the crystals from the surface is expected to occur during the composite preparation despite the DDM process being fast and thus lead to changes in the composition of the glass matrix. Complete dissolution of the crystals is thus expected when using crystals with smaller sizes than the ones used in this study.

**Figure 3:**
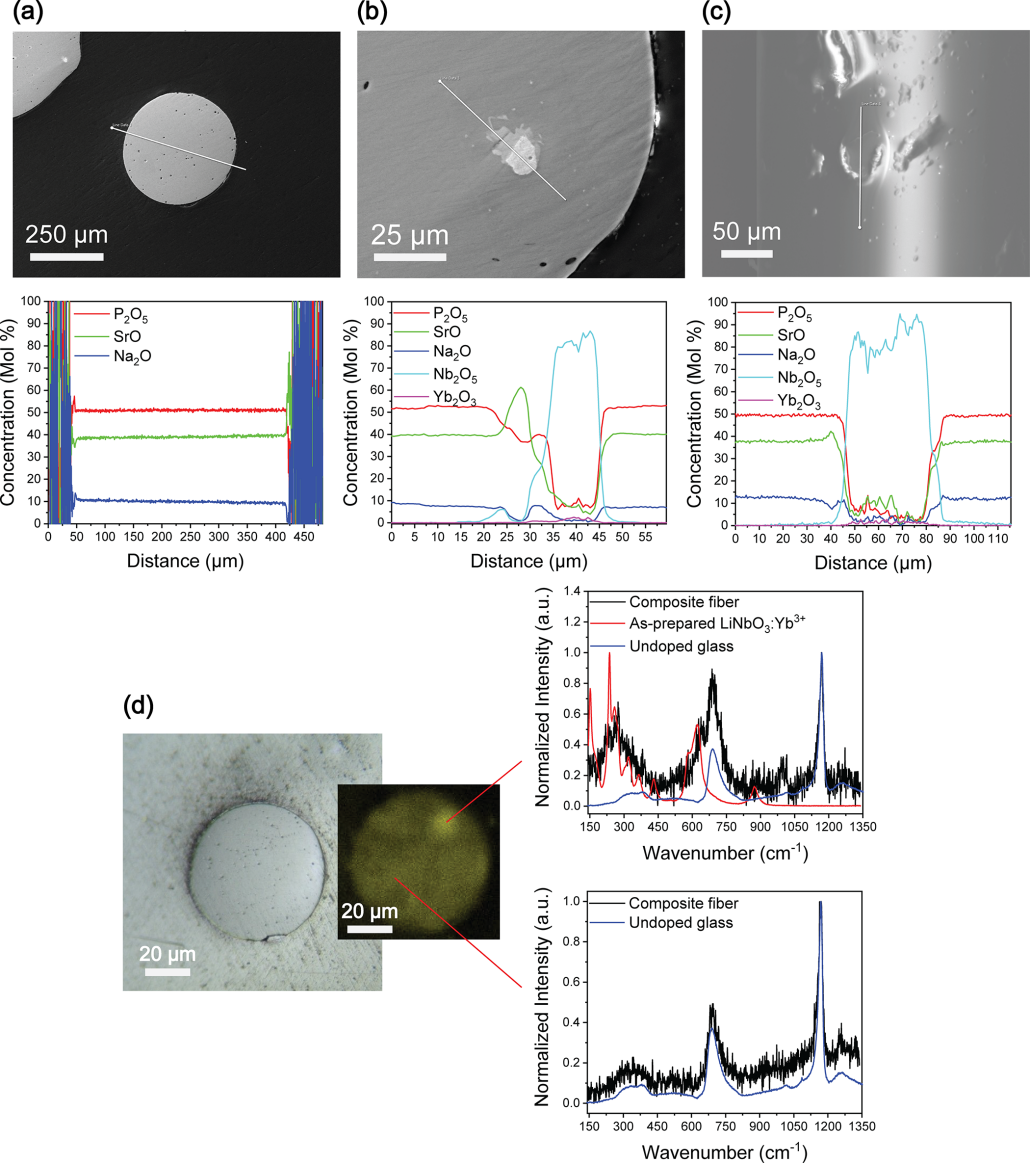
Structural analysis of the composite fibers with embedded LiNbO_3_:Yb^3+^ crystals. (a) SEM image of the composite fiber cross-section with a line scan. (b) SEM image of the composite fiber cross-section showing a crystal found inside the fiber with line scan. (c) SEM image of the composite fiber surface showing a crystal found on the surface of the fiber with line scan. (d) Microscope image (×10) of the fiber cross-section and its micro-Raman spectrum mapping of the intensity at 270 cm^–1^ that corresponds to the most intense micro-Raman band from the LiNbO_3_:Yb^3+^ crystal and micro-Raman spectra collected from two different spots (at highest intensity and at a random spot elsewhere).

Based on its spectroscopic properties, the composite prepared with the Yb_2_Si_2_O_7_ crystals seems to be the most promising. However, the composites tend to break easily upon cooling. Cracks can be seen in the SEM image presented in Figure 2e. The cracks appear to originate from the crystals and propagate into the glass, likely caused by the mismatched thermal expansion coefficients of the crystals (∼4 × 10^–6^/°C) and the glass (∼10 × 10^–6^/°C).^67,68^ Since the crystals do not dissolve in the glass, stress at the glass interface is probably high, leading to fragile composites. These cracks could be an issue, especially when preparing preforms that need to be at least 6 cm long and 1 cm in diameter to allow drawing fibers. However, for composites with embedded CaWO_4_:Yb^3+^ and LiNbO_3_:Yb^3+^ crystals, which have higher thermal expansion coefficients of ∼4–15 × 10^–6^/°C and ∼10–20 × 10^–6^/°C,^69,70^ respectively, cracks are not observed. This suggests that the better match in thermal expansion coefficients between these crystals and the glass reduces interfacial stress and improves the mechanical integrity of the composites. Therefore, composite preparations were carried out with the LiNbO_3_:Yb^3+^ crystals. Composites were fabricated with various amounts of crystals in order to get the most transparent composites with no cracks (see Figure S4a,b for the picture of the composites prepared with different amounts of crystals and their corresponding transmittance spectrum). As expected, the larger the amount of crystals, the least transparent the composites. As the composites did not break upon cooling, a crack-free preform with homogeneous dispersion of crystals was successfully prepared with 1 wt % of crystals, which corresponds to 0.01 mol % of Yb_2_O_3_ in the composite (see Figure S5 for the picture of the preform). This composite preform was drawn into fibers with diameters of 300 and 600 μm. No variation in the glass composition was induced during the drawing process, as evidenced using SEM coupled with EDS (Figure 3a). Evidence of the survival of LiNbO_3_:Yb^3+^ crystals during the drawing process is shown in Figure 3b and c. LiNbO_3_:Yb^3+^ crystals were seen not only in the fiber cross-section but also on the surface of the fiber. The white area in the SEM images corresponds to a high concentration of niobium, as confirmed by the line scan analysis. As observed in bulk samples, Sr-rich crystals are still seen at the glass–LiNbO_3_:Yb^3+^ crystal interface. Neither their size nor number seems to change after the drawing process. These additional crystals did not prevent the composite preform to be drawn into composite fibers. The presence of the LiNbO_3_:Yb^3+^ crystals inside the fiber was also confirmed by mapping of the micro-Raman signal at 270 cm^–1^, which is only seen in the micro-Raman spectrum of the LiNbO_3_:Yb^3+^ crystals (Figure 1b). The mapping of the fiber cross-section in Figure 3d reveals a distinct region with a high intensity of the signal at 270 cm^–1^. The micro-Raman spectrum measured in this region corresponds to that of the as-prepared crystals, whereas the micro-Raman spectrum of the region with low intensity corresponds to the signal from the glass matrix. It is clearly shown here that mapping of the micro-Raman signal at 270 cm^–1^ can be used to detect the presence of LiNbO_3_:Yb^3+^ crystals located beneath the glass matrix.

**Figure 4:**
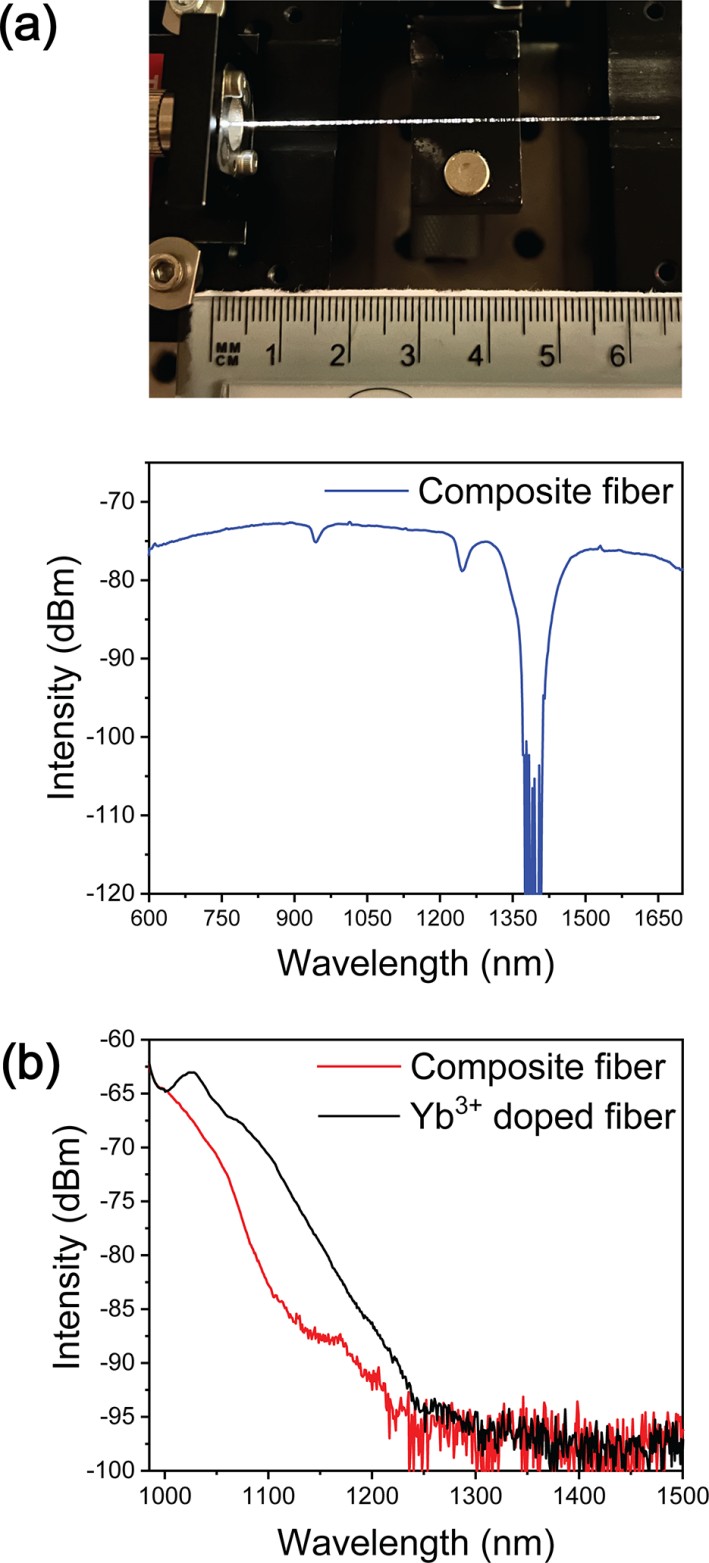
Spectroscopic analysis of the composite fibers with embedded LiNbO_3_:Yb^3+^ crystals. (a) Picture of the composite fiber guiding white light and its output intensity spectrum. (b) Emission spectra of the composite fiber (λ_exc_ = 980 nm).

Despite the presence of LiNbO_3_:Yb^3+^ crystals in the fiber, light can still propagate along its length, as evidenced by the output intensity spectrum and the picture of white light coupled into the fiber (Figure 4a). Scattering can be seen due to not only the presence of the crystals in the fiber but also various defects on the surface of the fiber. Indeed, one should remember that uncoated single-core fibers were drawn. Surface defects, such as microcracks, are thus expected on the surface of the fibers. Here, we demonstrate that despite the low amount of crystals (and so of Yb_2_O_3_), emission at ∼1 μm could be obtained from the composite fiber when pumped at 980 nm (Figure 4b). This emission band is similar to the one measured from a commercial Yb^3+^-doped fiber (Yb-10/125-1.6-PM) of the same length (3 cm).

## Conclusions

In summary, novel fibers are obtained from phosphate glass with embedded Yb^3+^-doped crystals. While dissolution of crystals, associated with the diffusion of Yb^3+^ ions into the glass matrix, is expected to occur when adding the crystals into the glass batch prior to melting, the crystals survive the melting process. Crack-free composite preforms prepared with LiNbO_3_:Yb^3+^ crystals can be successfully drawn into composite fibers. Light propagation in the composite fibers and emission from the composite fibers, despite their low amount of Yb_2_O_3_, are demonstrated. Adding crystals to the glass melt prior to quenching is confirmed to be an effective approach to integrate various crystals into different glass matrices. For the first time, we demonstrate that micro-Raman spectroscopy can be used to confirm the survival and presence of crystals beneath the fiber surface, which cannot be seen with neither an optical microscope nor a SEM.

The feasibility of drawing fibers from composite preforms is clearly demonstrated, highlighting the potential of these new composite fibers as a promising platform for advanced lasing applications.

## Supporting Information Available

Additional experiments and analyses, specifically XRD diffraction patterns of the as-prepared LiNbO_3_:Yb^3+^ crystals (Figure S1), flow diagram illustrating the synthesis of the composites using the DDM and remelt method (Figure S2), structural and spectroscopic properties of the composites prepared using the remelt method (Figure S3), images of composites prepared with varying LiNbO_3_:Yb^3+^ crystal content and their transmission spectra (Figure S4), and a photo of the composite preform prepared with the LiNbO_3_:Yb^3+^ crystals (Figure S5) (PDF)
