# Supplementary material for: A New Generation of Fibers Based on Yb3+-Doped Crystals Embedded in Phosphate Glass
Source: ACS Omega. 2025 Apr 17;10(16):16430–9. doi: 10.1021/acsomega.4c10954 (PMC12044438; doi:10.1021/acsomega.4c10954)
Supplement: Supplementary file 1 — ao4c10954_si_001.pdf [file ao4c10954_si_001.pdf]

A NEW GENERATION OF FIBERS BASED ON YB^{3+} DOPED CRYSTALS EMBEDDED IN PHOSPHATE GLASS

Natalia Vakula[*,†], Matiss Bardins[†], Khaldoon Nasser[†], Bartosz Bondzior[‡], Catherine Boussard- Plédel[+], Johann Troles[+], Laeticia Petit[†]

[†]Photonics Laboratory, Tampere University, Korkeakoulunkatu 3, 33720, Tampere, Finland

[‡]Institute of Low Temperature and Structure Research, Polish Academy of Sciences, Okólna 2, 50-422 Wroclaw, Poland

[+]Univ Rennes, CNRS, ISCR [(Institut des Sciences Chimiques de Rennes)] – UMR 6226, F-35000 Rennes, France

The initial synthesis of the $LiNbO_3:Yb^{3+}$ crystals followed the procedure outlined in D.H. Piva *et al* study.[1] The formation of crystals occurred through a solid-state reaction using an alumina crucible, incorporating 5 at% of Yb^{3+}. First, the temperature was raised to 950 °C over a span of 2 hours, with a heating rate of 3 °C per minute. However, XRD analysis shows a secondary crystalline phase (Figure S1). Following the initial synthesis procedure, the synthesis duration was extended to 4 hours, yet the results remained unchanged. Subsequently, the synthesis temperature was increased to 1200 °C with the aim of achieving pure crystalline phases, however, a secondary phase persisted. The efforts to concurrently increase both temperature and duration of heat treatment did not lead to successful synthesis of pure phase of $LiNbO_3:Yb^{3+}$. In a further attempt, the content of Yb^{3+} was reduced to 2.5 at%. However, a 0.5 g batch evaporated under these temperature conditions. To obtain a pure crystalline phase of $LiNbO_3:Yb^{3+}$, it was necessary to increase the batch size to 10 g. The XRD pattern of this crystal corresponds well to standard Powder Diffraction File entry 04-002-5061 of $LiNbO_3$. No shift of the diffraction peaks is observed due to the low concentration of Yb^{3+} incorporated in $LiNbO_3$.

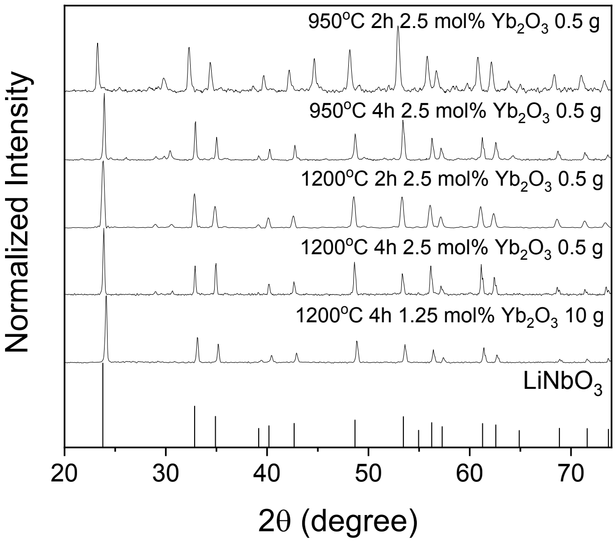

Figure S1. XRD diffraction patterns of the as-prepared crystals compared to the standard Powder Diffraction Files of $LiNbO_3$ (04-002-5061)

$CaWO_4:Yb^{3+}$, $Yb_2Si_2O_7$, and $LiNbO_3:Yb^{3+}$ crystals were synthesized using the solid-state reaction. Phosphate glass with the composition (in mol%) $50P_2O_5$ - 40SrO - $10Na_2O$ was prepared using the standard melt-quench method. The composites were prepared using 2 methods. In the remelt approach, the glass was first melted and then crushed into powder which was mixed with 5 wt% of the crystals. The mixture was melted at 950 °C for 7 minutes. After melting, the mixture was quenched onto a steel plate and annealed at 400 °C for 6 hours. The Figure S2 presents a flow diagram summarizing the glass preparation steps using the direct doping method (DDM) and the remelt method.

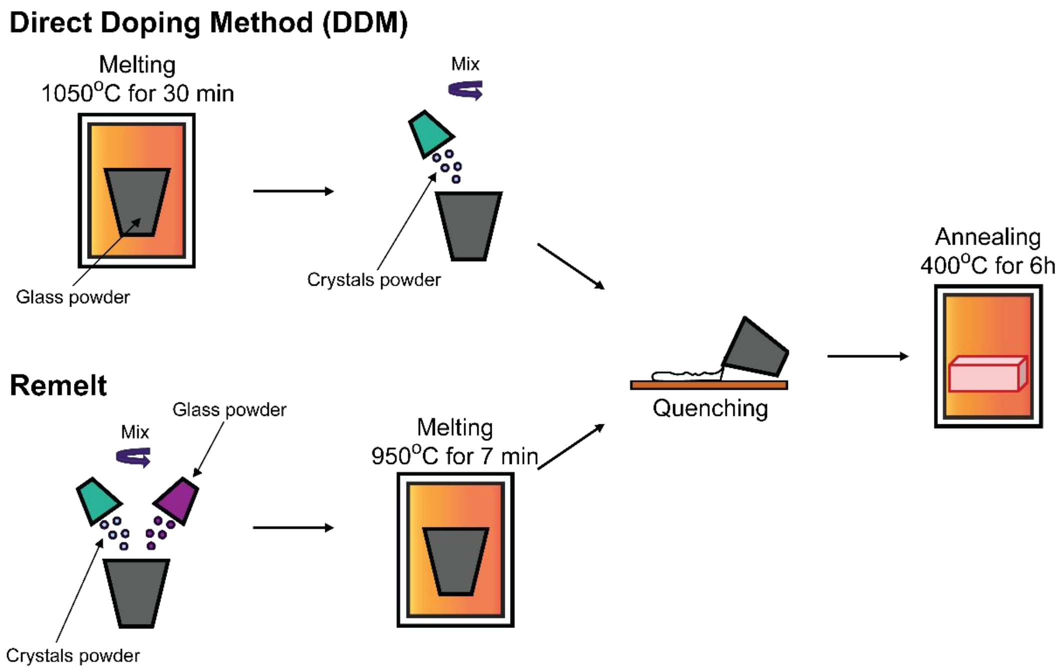

Figure S2. Flow diagram illustrating the synthesis of glass using the DDM and remelt method

In the remelt method, the crystal did not survive the remelt, meaning that they decomposed during the remelt process. This is evidenced from the changes in the shape of the emission band around 1 µm under excitation at 915 nm, after embedding the crystals in the glass matrix. The shape of the emission band of the composite is similar to that of Yb^{3+} doped glass as evidenced in Figure S3a. The partial/total dissolution of the crystals could be seen in the composites in the microscope images in Figure S3b. Furthermore, no micro-Raman peaks of the crystals could be detected in these composites confirming that the crystals likely dissolved completely into the glass matrix.

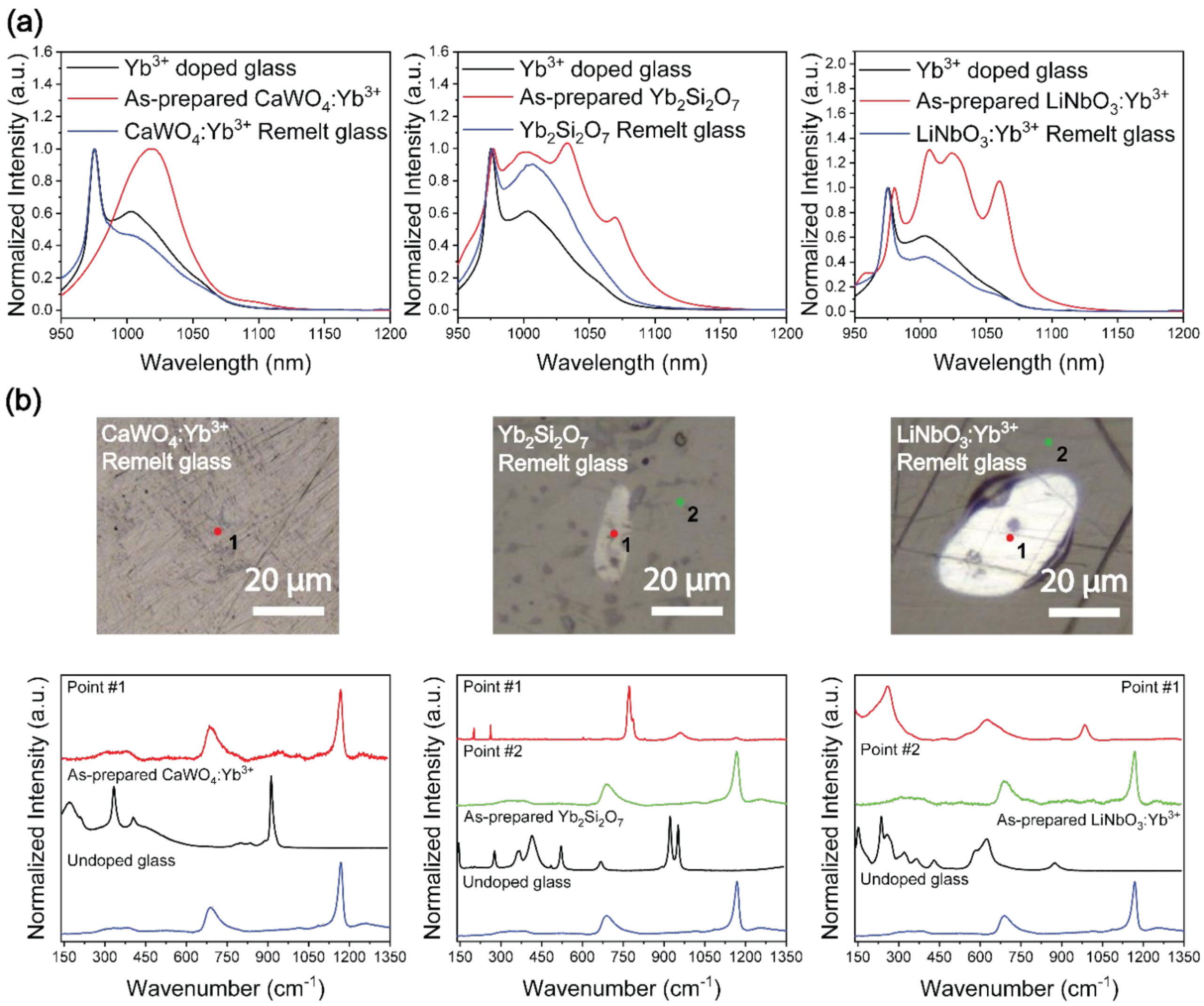

Figure S3. Structural and spectroscopic properties of the composites $CaWO_4:Yb^{3+}$, $Yb_2Si_2O_7$, and $LiNbO_3:Yb^{3+}$ crystals prepared using the remelt method. (a) Emission spectra of the remelt composites with embedded $CaWO_4:Yb^{3+}$, $Yb_2Si_2O_7$, and $LiNbO_3:Yb^{3+}$ crystals (λ_{exc}=915 nm). (b) Microscope image (x50) and micro-Raman spectra of the composites with embedded $CaWO_4:Yb^{3+}$, $Yb_2Si_2O_7$, and $LiNbO_3:Yb^{3+}$ crystals prepared using the remelt method.

In order to obtain transparent glass composites, the composites prepared with the DDM were prepared with various amounts of $LiNbO_3:Yb^{3+}$ crystals. Pictures of the composites are shown in Figure S4a. Figure S4b shows their transmission spectra from 200 to 1700 nm.

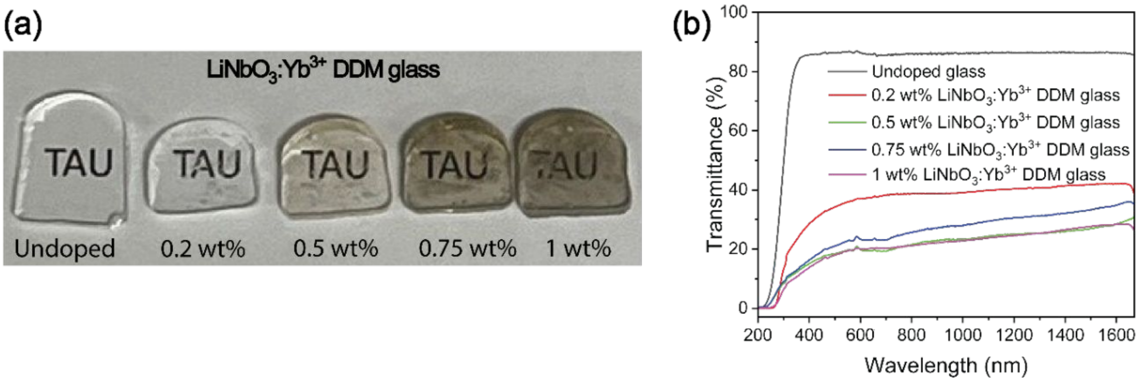

Figure S4. (a) Picture of the composites prepared with different amounts of $LiNbO_3:Yb^{3+}$ crystals using the DDM. (b) Transmission spectra of the composites.

A composite preform was successfully prepared with 1 wt% of $LiNbO_3:Yb^{3+}$ crystals. A picture of the composite preform is presented in Figure S5 showing the homogeneous dispersion of crystals.

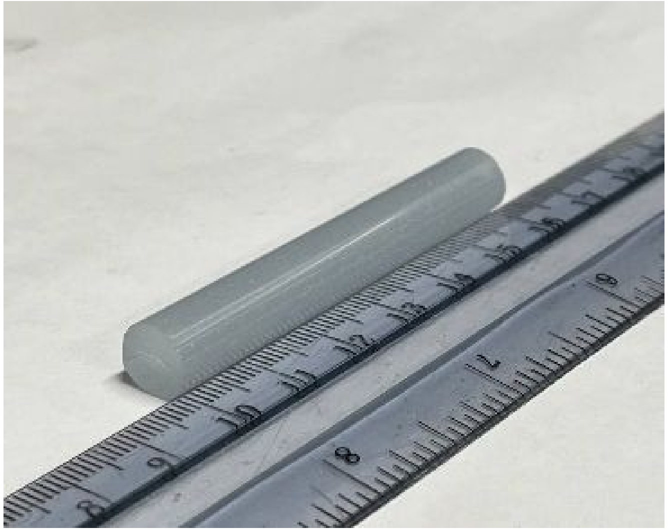

Figure S5. Photo of the preform doped with $LiNbO_3:Yb^{3+}$ crystals.

REFERENCES

(1) Piva, D. H.; Biz, H.; Piva, R. H.; Morelli, M. R. Influence of Oxidant and Fuel on the Powder Characteristics of LiNbO3 Synthesized by Combustion Method. *Bull Mater Sci* **2017**, *40* (1), 101–106. https://doi.org/10.1007/s12034-016-1350-4.
